# COVID‐19 and the adaptive evolution of genetic counseling

**DOI:** 10.1002/jgc4.1571

**Published:** 2022-03-18

**Authors:** Nathan C. Hassel, Adel D. Gilbert, Bassem A. Bejjani

**Affiliations:** ^1^ Metis Genetics Addison Texas USA; ^2^ Wake Forest University Graduate School of Arts and Sciences Winston‐Salem North Carolina USA

**Keywords:** COVID‐19, genetic counseling, genetic services, service delivery models, telemedicine

## Abstract

Emerging diseases such as the Coronavirus Disease (COVID‐19) have exposed severe weaknesses in the United States and global health. Healthcare systems have struggled and are still severely challenged and strained by this pandemic. It is clear that additional resources are needed to support healthcare providers in managing this and future pandemics. Genetic counselors can play an important supporting role in this fragile ecosystem because their comprehensive and broad training makes them uniquely qualified to meet many of the challenges that arise when healthcare workers and patients are faced with novel diseases. This paper describes the recent involvement of a telegenetic counseling company (Metis Genetics) in communicating and explaining COVID‐19 serum antibody results to patients and physicians. This experience demonstrates how genetic counselors may be called upon to play a vital supporting role in the management of infectious disease pandemics. From May 2020 to July 2020, our genetic counseling telegenetics team was asked to provide support to more than 1,580 patients who underwent serum COVID‐19 antibody testing and to educate their healthcare providers on the performance properties of this new test. The genetic counselors were able to utilize their expertise to convey test results, information on Center for Disease Control and Prevention (CDC) recommendations, COVID‐19 fact‐based evidence, to provide psychological support and reassurance to patients, and to respond to providers questions about the test. This experience suggests that the genetic counselors’ skillset that has allowed the profession to continuously evolve can also be used in the management of pandemics by communicating directly with the public, supporting other healthcare workers, and assisting individual patients and families navigate the many medical and psychological issues caused by such events.

## 1 A MORE COMPLEX WORLD

The world is becoming more complex. Human behavior is a major cause to an ever‐accelerating number of novel disease pandemics (H1N1 in 2009, MERS 2012, Ebola in 2013, Zika in 2016, and Coronavirus Disease [COVID‐19] in 2019), as humans encroach on wild animal habitats, crisscross the world, and inhabit denser cities, such global pandemics will continue to threaten humankind (Roche et al., 2020). In addition to these threats, emerging diseases such as COVID‐19 have exposed vulnerability in the United States and global health, such as shortages of personal protective equipment (PPE), availability of ventilators and intensive care beds, and limited testing (Emanuel et al., 2020; Tanne et al., 2020). Healthcare systems and providers must evolve and adapt to meet these new challenges. Genetic counselors can play an important supporting role in this fragile ecosystem.

Genetic counselors have comprehensive and broad training that makes them uniquely qualified to meet the challenges of a world besieged by novel diseases by utilizing their skillsets of communicating complex ideas, risk assessment, conveying ambiguous data, empathetic interpersonal communication, and facilitating adaptation to changing scientific information (Everett et al., 2014; Rabideau et al., 2016). The many roles of a genetic counselor have continued to evolve since the genesis of the specialty over 60 years ago, and further adaptation to this more complex world is essential (Baty, 2018).

## 2 THE EVOLUTION OF A SPECIALTY

The genomic revolution has transformed a once esoteric discipline into an essential component of today's health care. The proportion of the population that requires genetic counseling has steadily increased over the past 60 years and accelerated in the last 10 (Hooker et al., 2020). The role of genetic counselors has continued to broaden, partially due to the versatility of their core skillset and competencies, and partially due to our expanded understanding of the genetic basis of human disease. While genetic counselors primarily practiced in prenatal and pediatric specialties in the first years of the profession, today's practitioners find themselves playing crucial roles in oncology, cardiology, neurology, laboratory medicine, pharmacology, and other specialties (National Society of Genetic Counselors, 2021). Genetic counselors have also assumed important responsibilities beyond the clinic, including research, education, commercial laboratories, telehealth, public policy, and public health, to name a few (Abacan et al., 2019). Here, we report on our own recent experience with COVID‐19 and demonstrate that genetic counselors may also be called upon to play a vital supporting role in the management of disease pandemics.

## 3 COVID‐19 AS AN ENVIRONMENTAL STRESS FORCING ADAPTATION

The start of the COVID‐19 epidemic in the United States can be traced to January 19, 2020, when a 35‐year‐old man presented to an urgent care clinic in Snohomish County, Washington, with a 4‐day history of cough and subjective fever (Holshue et al., 2020). Due to the rapid spread of COVID‐19 in the United States, the limited availability of COVID‐19 testing, and the lengthy authorization process, the US Food and Drug Administration (FDA) moved to expand testing capacity by eliminating a requirement that advanced laboratories obtain prior FDA authorization before using their own, laboratory‐developed tests (LDTs) on February 29, 2020 (Sharfstein et al., 2020). By early March, with an increasing number of COVID‐19 cases being diagnosed across the United States, states imposed various levels of safety measures, severely restricting citizens’ mobility, which incidentally, negatively affected elective health care. While emergency rooms and in‐patient hospital services were being taxed beyond capacity, an aggregate trend of 40% decrease in outpatient visits was noted across the United States in the first week of March 2020 (Chatterji & Li, 2021). Parallel and contrasting with this drastic decrease in routine and non‐urgent medical care, laboratories engaged in SARS‐CoV‐2 testing saw a surge in demand that spurred a flurry of new tests utilizing existing technology.

In April 2020, a clinical diagnostic laboratory approached our telegenetics team with a proposal to assist in managing patient and provider inquiries regarding the results from their newly designed SARS‐CoV‐2 serum antibody LDT panel. These patient samples (adults only) were collected at facilities in California and Texas already providing phlebotomy services. The analysis was performed at the clinical laboratory's testing facility in San Diego.

The genetic counselors were able to utilize their expertise to convey positive, negative, and inconclusive antibody test panel results to patients. It is important to note that the genetic counselors were available to switch into this new role due to experiencing a similar decrease in patient volume as reported across the United States (Chatterji & Li, 2021), and they had the skillset necessary to comprehend and communicate these test results. With genetic counselors assuming this new responsibility, providers were able to devote their time and skills to treating patients.

Training of the genetic counselors included a half‐day videoconference with reviews of immunology, laboratory testing, the testing platform and performance as well as the current guidelines for COVID‐19 protocols established by the Center for Disease Control and Prevention (CDC). The counselors also held weekly video conferences with the clinical laboratory's staff for administrative and scientific updates. Patients and physicians contacting the clinical laboratory with questions regarding test results were directed to our telegenetics team. Four genetic counselors and one genetic counselor aide supported these patients by providing information and counseling on the telephone. The genetic counselors educated patients on differences between diagnostic and screening tests, explained false positives and false negatives, and the differences between positive and negative predictive values. The most frequent question received by the genetic counselors was requesting further explanation into a patients’ inconclusive antibody results. Patients often expressed their frustration and confusion in their attempts of interpreting their ambiguous results. The genetic counselors were able to use their empathetic interpersonal communication skills to express how they understood the patients’ anxiety and frustrations, allowing the genetic counselors to immediately build rapport. The genetic counselors utilized their knowledge of immunology and laboratory technologies to convey the patients’ results in a simplified manner. This newly formed trust allowed the genetic counselors to then highlight the importance of adhering to the CDC’s recommendations, and explain the clinical laboratory's retesting protocols. The compassionate support demonstrated throughout these phone calls allowed the patients to make rational follow‐up decisions. Other common questions received by the genetic counselors included quarantine protocols, infection risks, and antibody development. It is important to note that much of this information regarding COVID‐19 was still being studied. Genetic counselors used fact‐based evidence during a time where much remained to be discovered about COVID‐19. From May 2020 to July 2020, the genetic counselors were able to provide support to more than 1,580 patients. This experience highlights just one of the numerous evolutionary advancements genetic counseling has taken, as the profession has continued to broaden and positively impact today's healthcare system. This experience provides insight into how genetic counselors may use their skillset to expand their professional roles in other ways.

## 4 A NEW FUTURE FOR GENETIC COUNSELING

According to the National Society of Genetic Counselors (NSGC) code of ethics, the genetic counselor's role is to ‘seek out and acquire balanced, accurate and relevant information required for a given situation’ and to ‘continue their education and training to keep abreast of relevant guidelines, regulations, position statements, and standards of genetic counseling practice’. (NSGC code of Ethics, 2017). Genetic counselors, among healthcare professionals, combine knowledge of basic science, human genetics and genomics, epidemiology, and psychology with clinical skills in counseling, medical genetics, risk assessment, education, empathetic interpersonal communication, and psychosocial counseling. They are adept at conveying complicated concepts clearly in layman's terms. They are skilled at communicating emotionally difficult news to vulnerable individuals. They are trained to offer compassionate support to patients dealing with life‐altering news and help them to navigate into an unknown and often unknowable future. These qualities that have guided the evolution of genetic counseling into many new specialty areas make genetic counselors competent to play prominent supporting roles in the fight against emerging and novel diseases.

While it may not be initially intuitive to utilize genetic counselors in this way, genetic counselors’ skills can be used to communicate directly with the public, to support other healthcare workers, and to assist patients and families during future pandemics. As we face new threats, genetic counselors are ideally suited to play a central role in addressing these complex issues.

Healthcare specialties requiring genetic counseling have continued to increase over the past 60 years, as the profession grew to include counseling chromosome abnormalities, hemoglobinopathies, phenylalanine hydroxylase deficiency, and Tay‐Sachs disease among countless other genetic diseases (Bansal et al., 2010; Loader et al., 1991; Regier et al., 2017). Since the completion of the Human Genome Project (HGP) in 2003, the field of genetics and genomics has evolved to impact all areas of medicine; the role of the genetic counselor has expanded right along as demonstrated by the evolution of genetic counseling from a historical focus on rare Mendelian diseases to a broader practice of personalized medicine (Abacan et al., 2019; Hooker et al., 2020; Lander, 2011; Shelton & Whitcomb, 2015). It is, therefore, only natural for genetic counseling to address the evolving nature of viral pandemics, the emergence of new strains, the role that variants play in increased transmissibility of the virus, its potential resistance to vaccination efforts, and the possible relationship of a persons’ genetic make‐up with their susceptibility to infection or their propensity to have a more or less severe clinical course. This advancement in the profession has already begun, as genetic counselors were redeployed to new roles involving patient support, palliative care and COVID‐19 research (Ahimaz et al., 2020; Luu, 2020; Wagner et al., 2021). Therefore, the training of the next generation of genetic counselors ought to address this evolving field. One can imagine a new specialty in genetic counseling that focuses on the genetics of infectious diseases, immunology, public health, and epidemiology. Based on the experience of these genetic counselors, programs may want to consider preparation for the management of infectious diseases and pandemics. Training programs should ensure graduates are aware of the broad public health role genetic counselors may play in the future. Optimally, students should understand how infectious diseases can impact the population and how their skill set, translating complex information into lay terms, may be utilized effectively.

The Practice‐Based Competencies (PBCs) were created by the Accreditation Council for Genetic Counseling (ACGC) to summarize the proficiencies necessary for successful practice of entry‐level genetic counselors (Doyle et al., 2016; Practice‐Based Competencies for Genetic Counselors, 2019). Past studies established that genetic counselors were able to successfully utilize their PBCs as their professional roles grew (Field et al., 2016). The re‐evaluation of the PBCs may benefit training and continuing education, as certain PBCs were utilized more often than others, as genetic counselors were redeployed in response to the pandemic (Wagner et al., 2021). Genetic counselors will continue to achieve prominent roles in the fight against new diseases and future pandemics, as well as other emerging roles in healthcare.

## AUTHOR CONTRIBUTION

All authors contributed to the drafting of the manuscript. All authors contributed significantly to the editing and iterating of the manuscript. All of the authors gave final approval of this version to be published and agree to be accountable for all aspects of the work in ensuring that questions related to the integrity of this work are appropriately investigated and resolved.

## COMPLIANCE WITH ETHICAL STANDARDS

### Conflict of interest

Nathan Hassel, MS, CGC is a consultant for Metis Genetics, LLC. Bassem Bejjani, MD is a consultant for and has options in Metis Genetics, LLC. Adel Gilbert declares that she has no conflicts of interest.

### Human studies and informed consent

No human studies were carried out by the authors for this manuscript.

### Animal studies

No non‐human animal studies were carried out by the authors for this manuscript.

### Data sharing and data accessibility

The data that support the findings of this study are available from the corresponding author upon reasonable request.
